# A deep learning predictive model for public health concerns and hesitancy toward the COVID-19 vaccines

**DOI:** 10.1038/s41598-023-36319-6

**Published:** 2023-06-06

**Authors:** Heba Mamdouh Farghaly, Mamdouh M. Gomaa, Enas Elgeldawi, Heba Askr, Yaseen A. M. M. Elshaier, Hassan Aboul Ella, Ashraf Darwish, Aboul Ella Hassanien

**Affiliations:** 1 Computer Science Department, Faculty of Science, Minia University, Minya, Egypt; 2 Faculty of Computers and Artificial Intelligence, IS Department, University of Sadat City, Sadat City, Egypt; 3 Department of Organic and Medicinal Chemistry, Faculty of Pharmacy, University of Sadat City, Sadat City, 32897 Menoufia Egypt; 4 Microbiology Department, Faculty of Veterinary Medicine, Cairo University, Giza, Egypt; 5 Faculty of Science, Helwan University, Helwan, Egypt; 6 Faculty of Computer and AI, Cairo University, Giza, Egypt; 7 Scientific Research Group in Egypt (SRGE), http://www.egypscience.net

**Keywords:** Biochemistry, Drug discovery, Microbiology

## Abstract

Throughout the pandemic era, COVID-19 was one of the remarkable unexpected situations over the past few years, but with the decentralization and globalization of efforts and knowledge, a successful vaccine-based control strategy was efficiently designed and applied worldwide. On the other hand, excused confusion and hesitation have widely impacted public health. This paper aims to reduce COVID-19 vaccine hesitancy taking into consideration the patient’s medical history. The dataset used in this study is the Vaccine Adverse Event Reporting System (VAERS) dataset which was created as a corporation between the Food and Drug Administration (FDA) and Centers for Disease Control and Prevention (CDC) to gather reported side effects that may be caused by PFIEZER, JANSSEN, and MODERNA vaccines. In this paper, a Deep Learning (DL) model has been developed to identify the relationship between a certain type of COVID-19 vaccine (i.e. PFIEZER, JANSSEN, and MODERNA) and the adverse reactions that may occur in vaccinated patients. The adverse reactions under study are the recovery condition, possibility to be hospitalized, and death status. In the first phase of the proposed model, the dataset has been pre-proceesed, while in the second phase, the Pigeon swarm optimization algorithm is used to optimally select the most promising features that affect the performance of the proposed model. The patient’s status after vaccination dataset is grouped into three target classes (Death, Hospitalized, and Recovered). In the third phase, Recurrent Neural Network (RNN) is implemented for both each vaccine type and each target class. The results show that the proposed model gives the highest accuracy scores which are 96.031% for the Death target class in the case of PFIEZER vaccination. While in JANSSEN vaccination, the Hospitalized target class has shown the highest performance with an accuracy of 94.7%. Finally, the model has the best performance for the Recovered target class in MODERNA vaccination with an accuracy of 97.794%. Based on the accuracy and the Wilcoxon Signed Rank test, we can conclude that the proposed model is promising for identifying the relationship between the side effects of COVID-19 vaccines and the patient’s status after vaccination. The study displayed that certain side effects were increased in patients according to the type of COVID-19 vaccines. Side effects related to CNS and hemopoietic systems demonstrated high values in all studied COVID-19 vaccines. In the frame of precision medicine, these findings can support the medical staff to select the best COVID-19 vaccine based on the medical history of the patient.

## Introduction

There are several approaches to develop a vaccine. They differ in the percentage of the virus used. According to World Health Organization (WHO)^1^, one approach uses the whole virus or bacterium such as whole-microbe approach, another approach uses just the part of the virus which triggers the immune system of the body such as the subunit approach or uses just the genetic material which provides the instructions needed for creating specific proteins such as the genetic approach (nucleic acid vaccine).

Some COVID-19 vaccines use a genetically modified form of messenger RNA(mRNA) such as Pfizer-BioNTech and Moderna^2^. Having a deep look at the configuration of COVID-19, it can easily be noticed that the surface of the virus has a spike-like configuration referred to as S glycoprotein. When such mRNA enters the body through COVID-19 mRNA vaccines, it orders the body cells of a recipient to produce a harmless fragment of S protein. On the other hand, other COVID-19 vaccines are considered to be vector-based vaccines such as AstraZeneca, Janssen, and Gamaleya which depend on the recombination of spike gene from SARS-CoV-2 into other viral vector such as an adenovirus. Viral vector- based vaccines of SARS-CoV-2 do not have any pathogenesis for SARS-CoV-2 and the viral vector virus.

The adjuvant subunit vaccines such as Novavax and Biological E are mainly designed based on using more immunogenic S glycoproteins of SARS-CoV-2 associated with adjuvant^3^. This type of vaccine produces antibodies and defensive white blood cells once your immune system recognizes the S proteins. Protein subunit COVID-19 vaccines do not use live viruses and hence cannot infect the body with the COVID-19 virus. Protein fragments also do not enter the nucleus of body cells, where the DNA is stored. Inactivated vaccines such as Sinopharm, Sinovac Biotech, and Bharat Biotech are widely used in China, India, and many developing countries. This type of vaccine depends on adjuvanted whole inactivated viruses.

Artificial Intelligence (AI) is a technique that allows a machine to replicate human behavior which aims to create a functional model of the human brain that is capable of making decisions based on learning^4^. ML is a branch of AI that makes use of statistical methods to provide machines the ability to learn and develop over time. It includes a wide range of applications, and other techniques have been created like clustering, Bayesian network, decision tree, and DL^5^. DL is a specific type of ML that imitates how our brain cells operate which inspired the concept of neural networks^6^.

DL has proved to be a promising AI Subfield in many real-life sectors including healthcare and drug discovery sectors. Recently, with the advancement of DL, extensive pharmaceutical industries move toward AI-based methods. DL models are available in many sizes and shapes, able to solve problems in an efficient manner that are too complex for conventional methods to handle^7^.

COVID-19 vaccines still gain many remarks and limitations starting from mild side effect to sever disaster effects. In the frame of the necessity of the vaccinations all over the world to combat COVID-19 disaster, the aim of the paper is to examine the relation between side effects of three COVID-19 vaccines and the type of patients. Herein we categorized patients into three classes (Died, Hospitalized and Recovered). By utilizing DL, we discovered the relation of side effects to every class.

Because of the necessity of understanding the side effect of the COVID-19 vaccine especially since it is an outbreak disease worldwide, This work aims to examine the relationship between the type of COVID-19 vaccine (3 vaccines) and the generated side effects related to them. This approach was implemented by utilizing a DL model. Herein we categorized patients into three classes (Died, Hospitalized and Recovered). By utilizing DL, we aim to discover the relation of side effects to every class.

The main contributions of this paper are summarized as follows:

- To the best of our knowledge, this is the first research that uses DL to predict the adverse effects a patient may experience after the COVID-19 vaccination, which will have a remarkable impact on the public health concerns related to COVID-19 vaccine.
- Such a study will guide the medical doctors and pharmaceutical companies to select the vaccine according to patient history. Furthermore, this study will add a more pharmacovigeleince information about such vaccines.
- The most important features which affect the performance of the proposed model are optimized using the “Pigeon algorithm”.

The current work tried to figure out an efficient predictive method of the adverse and side effects of the COVID-19 vaccine which by its role will provide a very useful tool facing the previously discussed post-COVID-19 vaccine-related confusing situation.

The structure of the paper is organized as follows. Section “Literature review” presents a review of related work; Sect. “Basics and background” gives a preliminary background. Section “The proposal model” presents the proposed framework. Section “Experimental results” discusses the results. Finally, Sect. “Conclusion” gives a conclusion of the paper.

## Literature review

This section gives a summary of the previous work on the investigated problem in this paper.

In the Chinese province of Hubei, Wuhan City had an outbreak of a new coronavirus in December 2019. The majority of the patients who were initially diagnosed were found near the “wet market,” which is a place where live animals are slaughtered and sold. The market may have served as an amplifying hotspot from which the virus quickly spread to territories and other regions of China as well as 213 countries worldwide^8^.

On February 11, 2020, the world health organization (WHO) defined this disease as COVID-19, an acronym for Coronavirus Disease 2019. Globally, 761,000 fatalities and 21.2 million confirmed cases have been documented as of August 17th, 2020^9^. The worst COVID-19 scenarios reportedly occur in the USA, India, Brazil, and Russia, where the number of confirmed cases has exceeded that of China. The current COVID-19 epidemic was classified as a "Public Health Emergency of International Concern" by the WHO on January 30, 2020, and a "pandemic" on March 11, 2020.

SARS-CoV-2 is a zoonotic beta coronavirus that is transmissible to individuals through spillover outbreaks. It is a member of the subgenus Sarbecovirus and the Orthocoronavirinae subfamily of the family Coronaviridae. The SARS-CoV-2 animal reservoir is expected to be bats, but another plausible intermediate animal host is still unknown. The virus is a spherical particle that is 70–90 nm in size^10^. Glycoprotein spikes that protrude from its surface bind to the cell's angiotensin-converting enzyme 2 receptor. The virus resembles a crown because of these spikes from which the name "Coronavirus” was derived.

Although this emerging virus has a far lower death rate (2.9%) than SARS-CoV (9.6%) and MERS-CoV (34.4%), its high transmissibility rate in comparison to other coronaviruses has raised concerns across the globe. In addition to being affected by underlying co-morbidity, which includes concurrent disorders such as diabetes, hypertension, cancer, cardiovascular disease, and chronic respiratory disease, the fatality rate of COVID-19 changes with age^11–13^.

The pandemic's introduction sparked a race to discover and develop a vaccine to create herd immunity and lessen COVID-19’s harmful consequences. The work being done to create a vaccine is currently established and proven to show results. Rollouts across countries have started after certain vaccination candidates produced respectable results^14^.

All vaccine candidates to reach worldwide distribution licensed approval should pass through the pre-clinical animal and laboratory-based experimentation, followed by 4 phases of clinical trials; Phase 1 trial, is conducted before candidate vaccines enter human clinical trials to assess the vaccine's safety, establish dosages, and early highlight any potential side or adverse effects in a limited sample of participants. Phase 2 trials start looking into efficacy on larger groups and continue to investigate safety. Few vaccines reach phase 3 trials, which are substantially involve hundreds or tens of thousands of patients. These studies are used to validate and evaluate the efficacy of the vaccine and examine whether any uncommon side effects only manifest in large populations. Phase 4 trials, the last stage, are carried out after receiving national regulatory permission and involve extended post-marketing surveillance in a large population. Not all vaccines that have been given domestic approval are in the 4th developmental stage. When granting emergency use authorizations, regulators in many nations follow their distinct legislation and timetables, relying on diverse forms of evidence from various clinical trial phases. Even before phase 3 studies were finished, some national regulators, particularly those in Russia and China, started licensing vaccines for (restricted or extensive) public use^15^.

Currently, about 330 COVID-19 vaccine candidates are under development of which; 194 are in the pre-clinical trials, 42 in the phase 1 trials, 44 in phase 2 trials, 40 in phase 3 trials, and 10 in phase 4 trials. It is worth to be mentioned that of the previously mentioned under-development vaccines, 24 are actually in use and being offered to the general population. All those vaccines can be categorized into 4 main categories; inactivated whole-cell vaccine, protein subunit vaccine, viral vector vaccine, and nucleic acid (RNA or DNA) vaccine. The most leading and commonly distributed ones are those produced by RNA PFIZER/BioNTech (Germany), MODERNA (USA), and viral vector JANSSEN/JOHNSON&JOHNSON (USA)^16^.

Tarik Alafif et al. in ref.^17^ have surveyed ML and DL-based research which conducted for the diagnosis of COVID-19. The authors looked as well at publicly available datasets that could be used. Their survey has shed light on most state-of-the-art approaches for ML and DL used and summarized potential challenges and future directions. M. Ali et al. in ref.^18^ presented another survey to study how ML deeply impacted understanding the Covid-19 pandemic. The survey focused on the virus diagnosis using X-ray images and CT scans. Future scenarios of the pandemic are also been provided in this survey.

Zhoe et al.^19^ adopted a CNN for COVID-19 CT testing. They examined the performance of different pre-trained models on CT images and concluded that their model can explore new visual indicators to help clinical physicians in further manual screening.

Hatmal et al. in ref.^20^, have analyzed several ML models to compare their accuracy in predicting the level of hospital care needed for patients diagnosed with Covid-19. They applied feature selection and oversampling techniques and their experimental results showed that age and gender are the most significant variables in the mentioned prediction problem. Patients are classified as if they need just a regular hospital admission or intensive care unit admission.

Bai et al.^21^. Collected CT scans from 1186 patients with either non-COVID pneumonia or RT-PCR-confirmed COVID pneumonia from 11 different hospitals in USA and China. They developed a deep learning model to discriminate between COVID and non-COVID pneumonia. The authors subsequently provided the model to radiologists and demonstrated that their model significantly improved radiologist diagnostic accuracy from 85 to 90% in distinguishing COVID pneumonia from non-COVID pneumonia.

Hernández-Pereira E. et al. in ref.^22^ presented a ML model that could predict whether a given Covid-19 patient is more likely to survive or die based on the patient’s medical history and demographic data. They used a dataset of confirmed and suspected infected patients in Mexico. They proved that their proposed model could identify high-risk patients and hence improve timely treatment and hospitalization. Chadagahttps et al. in ref.^23^ surveyed existing ML and DL methodologies and how they can improve our understanding of COVID-19 and avoid the outbreak of COVID-19.

Another study was conducted at King Fahad University Hospital, Dammam, KSA^24^. This study aims to automate COVID-19 diagnosis by integrating clinical patient data with chest X-ray images in a DL model. The data used contains a total of 270 patient records. The experiments were performed first with clinical data, second with the chest X-ray, and finally with both clinical data and the chest X-ray. This fusion is used to combine the clinical features and features extracted from images. The experimental results showed that their model improves diagnostic accuracy.

The vaccine development step including process legislation and regulation was considered by the whole world as “the light at the end of the COVID-19 dark tunnel”. Although the remarkable lifesaving role has been achieved through the globally adopted vaccine-based control strategy, the vicious vaccine development competition left the whole world confused.

As mentioned above, the literature is so rich with COVID-19 research. However, most of the work was concerned with the prediction and diagnosis of the COVID-19 infection, either by using the current condition and symptoms of the patient as features or the chest X-ray to diagnose the disease. To the best of our knowledge, this is the first research that uses DL to predict the adverse effects a patient may experience after COVID-19 vaccination.

## Basics and background

This section presents the preliminary for the algorithms used in this paper to help readers easily understand the methodology.

### Binary pigeon-inspired algorithm

The Pigeon algorithm is a bio-inspired optimizer that has been developed in 2014^25^. It is a population-based swarm intelligence algorithm that has been widely used and successfully applied to solve many optimization problems. Bio-inspired algorithms have always been tempting for researchers because of their ability to solve complex problems with extra-large search space such as non-deterministic polynomial problems. The Pigeon algorithm tries to improve the quality of the solutions by imitating the social behavior of a Pigeon swarm using a formulating a mathematical model based on their natural behavior^26^.

Homing behavior of Pigeons is derived from two operators: map and compass. Pigeons can sense the magnetic field of the earth, moreover, they use the sun’s altitude as a compass to constantly modify their direction to reach their home destination. When flying pigeons get closer to their home or destination, their need for the map and compass operator becomes less and less. The position *P*_*i*_ as well as the velocity *V*_*i*_ of each pigeon *i* are updated at each iteration *t*. This can be mathematically represented by the following equations^27,28^.

1 $$ V_{i} (t\, + \,1)\, = \,V_{i} \left( t \right).e^{ - Rt} \, + \,r\,\,and. \, \left( {P_{g} \, - \,P_{i} \left( t \right)} \right), $$

2 $$ P_{i} (t\, + \,1)\, = \,P_{i} \left( t \right)\, + \,V_{i} (t\, + \,1), $$

where *R* is a map and compass factor, while *rand* is a uniform random number in the range [0, 1], *P*_*g*_ is the global best solution.

**Figure 1:**
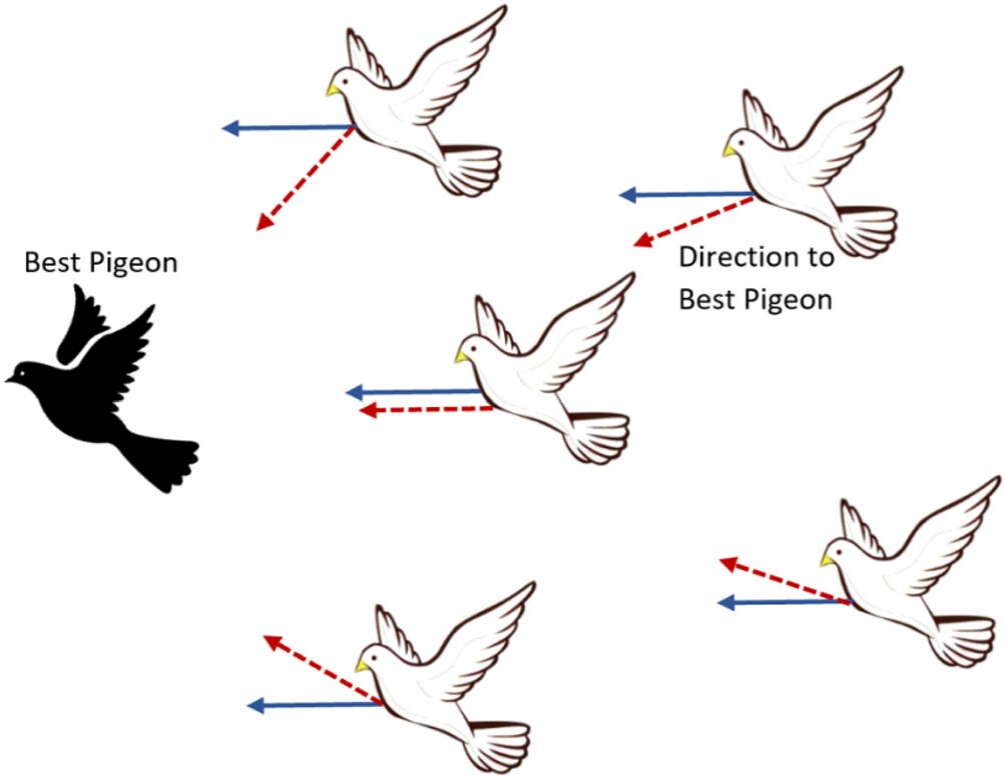
All pigeons adjust their position by following the best pigeon position.


At each iteration of the Pigeon algorithm, the pigeons depend on the map and compass operator to trace what so called the best Pigeon and hence modify their flying position, and the current flying direction of a given pigeon is represented mathematically by the first operand of Eq. (1) and is shown in Fig. 1 by the blue arrow. The flying direction of the best pigeon is denoted by the dotted red arrow and is represented mathematically by the second operand of Eq. (1). The next direction to which a given pigeon should navigate is represented by summing the two operands on Eq. (1). New flying positions of all pigeons are calculated at every iteration and modified accordingly using Eqs. (1) and (2). The Pigeon optimizer is listed in Algorithm (1).

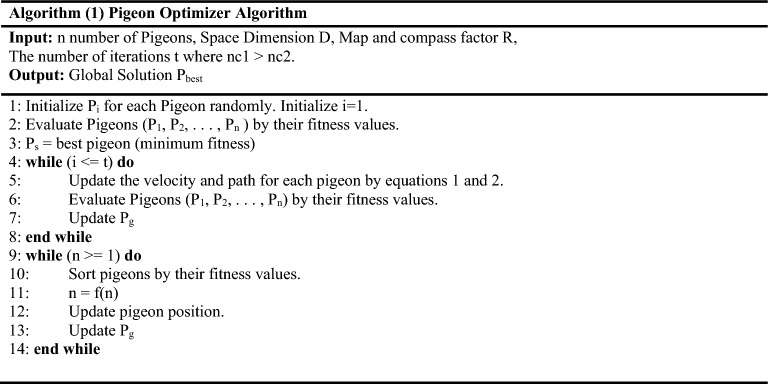


### Recurrent neural network

**Figure 2:**
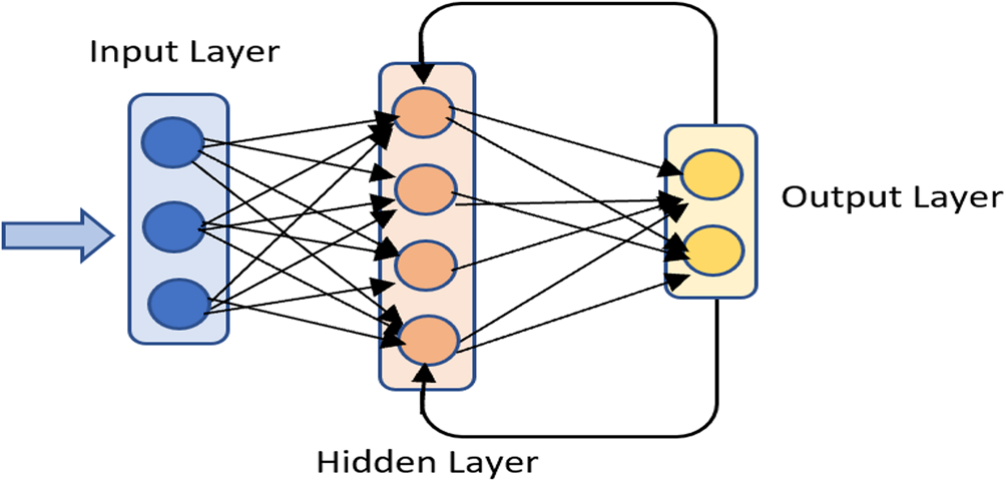
Recurrent neural network.

Recurrent Neural Network (RNN) is a special case of Neural Network where the output from a previous step is used back as an input to the next step. In most neural networks, inputs do not depend on outputs, but in some cases, the current prediction depends on previous prediction values. For example, when predicting the next word in a sentence, the words obtained from previous iterations are required and hence all previous words should be remembered to obtain the next word in the sequence. This issue is solved by using a hidden layer that remembers information obtained from past iterations (Refer to Fig. 2). In addition to hidden layers, RNN also uses backward loops throughout the computational process to feedback information into the network. Both hidden layers and backward loops give RNNs the ability to process sequential and temporal data.

## The proposed model

**Figure 3:**
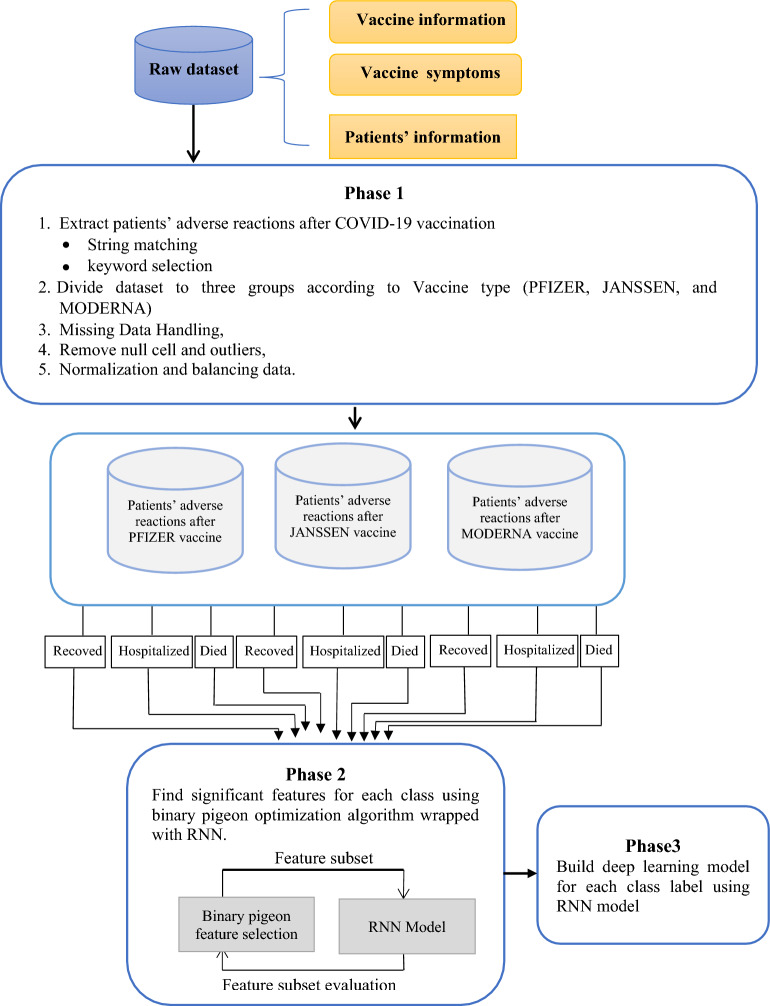
The architecture of the proposed model.

In this section, a discussion of the proposed model is presented. As shown in Fig. 3, the model undergoes three phases (1) data preprocessing, (2) feature selection Process, and (3) prediction phases. Each of these phases is explained in the following subsections after a brief description of the data used.

### Dataset description

The dataset used in the proposed model is the Vaccine Adverse Event Reporting System (VAERS) dataset^29^. It was created as a corporation between the Food and Drug Administration (FDA) and Centers for Disease Control and Prevention (CDC) to gather reported side effects that may be caused by PFIEZER, JANSSEN, and MODERNA vaccines. The dataset contains adverse reactions reported for several vaccines, not just SARS-CoV-2 vaccine. All vaccines other than COVID-19 vaccines were excluded from the current study. The dataset contains information about post-vaccination side effects. VAERS is used to let a patient report side effects experienced after vaccination. The dataset consists of three data files which are updated periodically with the most recent update date being referenced on the website. The dataset includes three files. One file is dedicated to patients’ demographic and medical history, the second file contains patients’ post-vaccination reactions, and the last file contains vaccine information. The dataset files have been merged according to the primary key ‘VAERS ID’ and only COVID-19 vaccinated patients’ data have been used. Table 1 presents the used variables and their descriptions.

**Table 1:** Dataset description.

Index	Features (inputs)	Description
**(**A) The patients’ information		
0	AGE_YRS	Age in years
1	SEX	Sex
2	L_THREAT	Life-threatening illness
3	OTHER_MEDS	Other medications
4	PRIOR_VAX	Prior vaccination event information
5	BIRTH_DEFECT	Congenital anomaly
6	OFC_VISIT	Healthcare provider or doctor clinic visit
7	ER_ED_VISIT	Emergency room care
8	ALLERGIES	Allergies to medications, food, or other products
9	VAX_DOSE_SERIES	Number of doses administered
10	DISABLE	Disability
11	COVID-19	Name of the diseases
12	SARS-COV-2 TEST POSITIVE	Positive test with the causative agent of COVID-19(SARS-CoV-2)
13	INTENSIVE CARE	Special hospital wards that provide treatment and monitoring for people who are very ill
(B) The patients’ adverse reactions after COVID-19 vaccination		
14	HEADACHE	Pain in your head or face that’s often described as a pressure that's throbbing, constant, sharp or dull
15	PYREXIA	Raised body temperature raised body temperature
16	DYSPNOEA	Shortness of breath or breathlessness,
17	FATIGUE	Overall feeling of tiredness or lack of energy
18	CHILLS	Feeling cold after being exposed to cold temperatures
19	PAIN	A warning sign in your nervous system that something is wrong
20	DIZZINESS	A term used to describe a variety of feelings
21	NAUSEA	The urge to vomit
22	PAIN IN EXTREMITY	Pain in areas of your body other than your head and torso, such as your arms,
23	ASTHENIA	Abnormal physical fatigue or lack of energy
24	MALAISE	General feeling of discomfort or illness whose exact cause is unknown
25	COUGH	Expel air from the lungs with a sudden sharp sound
26	INJECTION SITE PAIN	Common post vaccination symptoms
27	MYALGIA	Muscle aches and pain
28	HYPOAESTHESIA	Partial or total loss of sensation in a part of your body
29	CHEST PAIN	The most common heart problems
30	FEELING ABNORMAL	Tingling, prickling, or numbness anywhere on your body
31	RASH	An area of the skin that has been changed in texture or color and may look inflamed or irritated
32	CONDITION AGGRAVATED	To cause a patient's condition to deteriorate
33	CHEST DISCOMFORT	Sudden intense pain that appears to “tear” across the chest
34	ARTHRALGIA	Pain in a joint
35	PARAESTHESIA	A burning or prickling sensation that is usually felt in the hands
36	UNRESPONSIVE TO STIMULI	Not responding to some influence or stimulus
37	DIARRHOEA	The passage of three or more loose or liquid stools per day
38	PRURITUS	Itchy skin is an uncomfortable, irritating sensation
39	HEART RATE INCREASED	Rise in heart rate caused by exercise or a stress response
40	URTICARIA	Raised or puffy areas of the skin that itch intensely (hives)
41	FACIAL PARALYSIS	An inability to move the muscles of the face on one or both sides
42	SYNCOPE	Fainting or passing out. unconscious and go limp, then soon recover
43	TACHYCARDIA	Rise in heart rate caused by exercise or a stress response
44	PALPITATIONS	Having a fast-beating, or fluttering heart
45	HYPERHIDROSIS	Electrolyte imbalance
46	ERYTHEMA	Abnormal redness of the skin or mucous membranes due to capillary congestion
47	THROAT TIGHTNESS	Muscle tension dysphonia (MTD)
48	TREMOR	Involuntary, rhythmic muscle contraction leading to shaking body movements
49	BLOOD PRESSURE INCREASED	Hypertension disease
50	ANAPHYLACTIC REACTION	Severe, potentially life-threatening allergic reaction
51	LOSS OF CONSCIOUSNESS	Partial or total loss of perception of yourself and everything around you
52	DECREASED APPETITE	When your desire to eat is diminished
53	MUSCULAR WEAKNESS	Lack of exercise, ageing, muscle injury
54	FLUSHING	Become markedly red in the face and often other areas of the skin, from various physiological conditions
55	MOBILITY DECREASED	The partial or total loss of the ability to perform activities of daily living
56	INJECTION SITE ERYTHEMA	Swelling, redness (erythema), pain and itch at the site of injection can be a common
57	FEELING HOT	Due to many factors for example problem in thyroid or medication or underlying health condition
58	ABDOMINAL PAIN	Constipation, food allergies, lactose intolerance, food poisoning, and a stomach virus. Some other cases may be an abdominal aortic aneurysm, a bowel blockage, cancer, and appendicitis
59	INJECTION SITE SWELLING	Reaction to the needle or to the medicine that was injected
60	CEREBROVASCULAR ACCIDENT	It is considered the medical term for a stroke. A stroke can be defined as a blockage of the flow of the blood to a part of the brain or the rupture of a blood vessel
61	CARDIAC ARREST	The heart stops beating suddenly
62	LYMPHADENOPATHY	Swelling of lymph nodes which can be secondary to bacterial, viral, or fungal infections and autoimmune disease
(C) The patients’ classes (outputs)		
63	DIED	Died
64	HOSPITAL	Hospitalized
65	RECOVD	Recovered

Idiosyncratic adverse effects can affect a variety of organs, including the skin, muscle, liver, kidney, and heart, and some drugs/vaccines can cause more generalized hypersensitivity reactions. According to the severity of side effects, it is generally graded from 1 to 4. Grade 1 is very mild and grade 4 is very serious.

- GRADE 1 (Mild).
- GRADE 2 (Moderate).
- GRADE 3 (Severe).
- GRADE 4 (Potentially life-threatening).

This study is directed to figure out the grade of side effects in each type of COVID-19 vaccine mentioned in the current data set. Table 2 describes the organ or physiological system related to the side effect.

**Table 2:** Classification of patients’ adverse reactions after COVID-19 vaccination.

Feature	Classification
Headache	Central nervous system (CNS)
Pyrexia	CNS
Dyspnoea	Gastrio intestinal tract (GIT)/CNS
Fatigue	Autonomic nervous system (ANS)
Chills	CNS/ANS
Pain	CNS
Dizziness	CNS
Nausea	GIT
Pain in extremity	CNS
Asthenia	Blood/GIT/CNS
Malaise	ANS/CNS
Cough	Lung
Injection site pain	Blood, allergy
Myalgia	CNS/ANS
Hypoaesthesia	CNS/ANS
Chest pain	Respiratory
Feeling abnormal	CNS/ANS
Rash	Allergy, blood
Condition aggravated	CNS
Chest discomfort	Respiratory/ allergy
Arthralgia	Cardiovascular system (CVS)
Paraesthesia	CNS/ANS
Unresponsive to stimuli	CNS
Diarrhoea	GIT
Pruritus	Skin, allergy
Heart rate increased	Cardiovascular
Urticaria	Allergy
Facial paralysis	CNS
Syncope	CNS
Tachycardia	Cardiovascular/cardiac
Palpitations	Cardiovascular/cardiac
Hyperhidrosis	Blood
Erythema	Cardiovascular/cardiac
Throat tightness	Upper respiratory
Tremor	Blood/skin
Blood pressure increased	Cardiovascular
Anaphylactic reaction	Cardiovascular/allergic
Loss of consciousness	CNS
Decreased appetite	CNS
Muscular weakness	Neuromuscular disorders
Flushing	Blood vessels, endocrine
Mobility decreased	CNS, nervous
Injection site erythema	Vascular
Feeling hot	Endocrine
Abdominal pain	GIT
Injection site swelling	Blood/skin
Cerebrovascular accident	CNS
Cardiac arrest	Cardiovascular/cardiac
Lymphadenopathy	Blood

### Data preprocessing phase

From the investigated dataset, patient adverse reactions are considered in this study. The ‘SYMPTOM_TEXT’ field of the raw dataset includes the patient's overall medical history. By using string matching techniques, all existing medical conditions and pre-conditions of the patient have been extracted from the ‘SYMPTOM_TEXT’ field of the dataset. Each condition has been added as a separate binary field to the dataset, where ‘0’ denotes that the patient does not have this medical condition, and ‘1’ indicates that she/he suffers from such a condition. 49 reported medical conditions have been found in the ‘SYMPTOM_TEXT’ field. Examples of the included medical conditions are hypertension, diabetes, COPD, kidney disease, depression, and asthma. Reported COVID-19 vaccine adverse reactions are also included.

Some vaccine recipients had experienced some symptoms and died shortly after vaccination, others were reinfected with COVID-19, and some had experienced severe side effects and required hospital treatment admission to hospital facilities. Three different types of target classes for patient post-vaccination reaction analysis are considered. They are “death status”, “hospital admission status”, and “Recovered”. The three classes are not mutually exclusive.

For the VAERS dataset, data cleaning is performed by removing any missing values. Moreover, the dataset is not properly distributed. The hybrid sampling algorithm that combines over- and under-sampling techniques can be applied to the dataset to overcome this problem.

**Figure 4:**
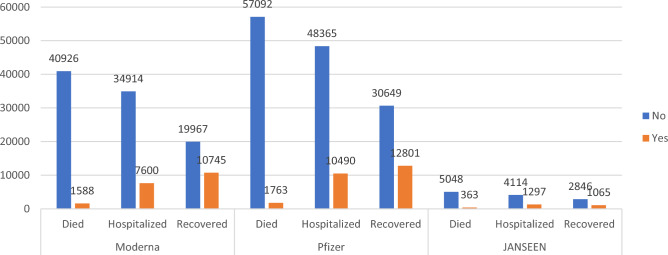
Class distribution for the patients’ adverse reactions after PFIEZER, JANSSEN and MODERNA vaccination.

Graphical demography of the target classes is given in Fig. 4 representing the distribution of individuals for each target class within the dataset for PFIEZER, JANSSEN, and MODERNA vaccines.

### Feature selection phase

Choosing the right features that can improve the accuracy of the DL model is a very important step in the classification process. Feature selection can be defined as the process of choosing a subset of features from a pool of original features that could influence the outcome the most. Feature selection improves predictive quality and performance, and the most important advantage of feature selection is the less computational time by the classification model. In the proposed model, the Pigeon algorithm has been used for the feature selection phase.

The Pigeon algorithm has been used during the feature selection phase to find the best features. The cost function used is given in Eq. (3). The solution is a subset of selected features from the original feature set in terms of True Positive Rate (TPR), False Positive Rate (FPR), and some features. With this fitness function, all features that do not affect the TPR or the FPR are eliminated as they will not change the quality of the solution. Equation (3) presents the formula used to evaluate the pigeon or solution fitness^30^.

3 $$\mathrm{F}=a\left(\frac{N}{TN}\right)+b \left(FPR\right)+c \left(\frac{1}{TPR}\right)$$

where *N* is the number of selected features, *TN* is the total number of features, and *a* + *b* + *c* = 1.

Tables 3, 4, and 5 indicate the best features selected by the Pigeon optimizer for PFIEZER, JANSSEN, and MODERNA vaccines respectively. It can be noticed that in case of PFIEZER vaccine some features are considered significant for all the three target classes “Death”, “Hospitalized”, and “Recovered” such as age, allergies, pain, 'intensive care'. On the other hand, some features are noticed to be specific for a certain target class such as 'tachycardia' feature for “Death” target class, 'feeling abnormal' feature for “Recovered” target class, and 'SARS-CoV-2 test positive' feature for “Hospitalized” class. Similarly, for JANSSEN, and MODERNA vaccines some features are common for the three patient target classes and other features are specific for each target class.

**Table 3:** The most significant features within the patient’s adverse reactions after PFIEZER vaccination for each target class.

No. of selected features		Selected features
Died	25	['AGE_YRS', 'SEX', 'L_THREAT', 'OFC_VISIT', 'ALLERGIES', 'HEADACHE', 'PAIN', 'DIZZINESS', 'ASTHENIA', 'INJECTION SITE PAIN', 'RASH', 'CONDITION AGGRAVATED', 'CHEST DISCOMFORT', 'PARAESTHESIA', 'UNRESPONSIVE TO STIMULI', 'SYNCOPE', 'TACHYCARDIA', 'ERYTHEMA', 'INTENSIVE CARE', 'LOSS OF CONSCIOUSNESS', 'FLUSHING', 'FEELING HOT', 'CEREBROVASCULAR ACCIDENT', 'CARDIAC ARREST', 'LYMPHADENOPATHY']
Hospitalized	33	['AGE_YRS', 'SEX', 'L_THREAT', 'OTHER_MEDS', 'PRIOR_VAX', 'ER_ED_VISIT', 'VAX_DOSE_SERIES', 'DISABLE', 'PYREXIA', 'DYSPNOEA', 'PAIN', 'DIZZINESS', 'NAUSEA', 'PAIN IN EXTREMITY', 'ASTHENIA', 'COUGH', 'INJECTION SITE PAIN', 'ARTHRALGIA', 'UNRESPONSIVE TO STIMULI', 'DIARRHOEA', 'PRURITUS', 'URTICARIA', 'FACIAL PARALYSIS', 'TACHYCARDIA', 'ANAPHYLACTIC REACTION', 'INTENSIVE CARE', 'DECREASED APPETITE', 'FLUSHING', 'ABDOMINAL PAIN', 'INJECTION SITE SWELLING', 'CEREBROVASCULAR ACCIDENT', 'LYMPHADENOPATHY', 'SARS-COV-2 TEST POSITIVE']
Recovered	30	['AGE_YRS', 'OTHER_MEDS', 'PRIOR_VAX', 'OFC_VISIT', 'ER_ED_VISIT', 'ALLERGIES','VAX_DOSE_SERIES', 'DISABLE', 'HEADACHE', 'PAIN', 'DIZZINESS', 'NAUSEA', 'INJECTION SITE PAIN', 'CHEST PAIN', 'FEELING ABNORMAL', 'RASH', 'UNRESPONSIVE TO STIMULI', 'PRURITUS', 'FACIAL PARALYSIS', 'SYNCOPE', 'THROAT TIGHTNESS', 'TREMOR', 'ANAPHYLACTIC REACTION', 'INTENSIVE CARE', 'DECREASED APPETITE', 'FLUSHING', 'FEELING HOT', 'ABDOMINAL PAIN', 'INJECTION SITE SWELLING', 'CEREBROVASCULAR ACCIDENT']

**Table 4:** The most significant features within the patient adverse reactions after JANSSEN vaccination for each target class.

No. of selected features		Selected features
Died	32	['AGE_YRS', 'SEX', 'OTHER_MEDS', 'PRIOR_VAX', 'OFC_VISIT', 'ER_ED_VISIT', 'ALLERGIES', 'VAX_DOSE_SERIES', 'HEADACHE', 'PYREXIA', 'FATIGUE', 'PAIN', 'NAUSEA', 'PAIN IN EXTREMITY', 'COUGH', 'HYPOAESTHESIA', 'FEELING ABNORMAL', 'RASH', 'DIARRHOEA', 'HEART RATE INCREASED', 'URTICARIA', 'FACIAL PARALYSIS', 'SYNCOPE', 'TACHYCARDIA', 'HYPERHIDROSIS', 'ERYTHEMA', 'LOSS OF CONSCIOUSNESS', 'DECREASED APPETITE', 'ABDOMINAL PAIN', 'INJECTION SITE SWELLING', 'CEREBROVASCULAR ACCIDENT', 'CARDIAC ARREST']
Hospitalized	24	['AGE_YRS', 'L_THREAT', 'OTHER_MEDS', 'BIRTH_DEFECT', 'OFC_VISIT', 'ALLERGIES', 'VAX_DOSE_SERIES', 'DISABLE', 'HEADACHE', 'PYREXIA', 'DYSPNOEA', 'DIZZINESS', 'NAUSEA', 'PAIN IN EXTREMITY', 'ASTHENIA', 'COUGH', 'CHEST DISCOMFORT', 'DIARRHOEA', 'PRURITUS', 'HEART RATE INCREASED', 'MUSCULAR WEAKNESS', 'INJECTION SITE ERYTHEMA', 'LYMPHADENOPATHY', 'SARS-COV-2 TEST POSITIVE'']
Recovered	36	['AGE_YRS', 'SEX', 'OTHER_MEDS', 'OFC_VISIT', 'ALLERGIES', 'DISABLE', 'FATIGUE', 'CHILLS', 'PAIN', 'NAUSEA', 'COVID-19', 'PAIN IN EXTREMITY', 'ASTHENIA', 'MALAISE', 'HYPOAESTHESIA', 'FEELING ABNORMAL', 'RASH', 'ARTHRALGIA', 'PARAESTHESIA', 'DIARRHOEA', 'HEART RATE INCREASED', 'URTICARIA', 'FACIAL PARALYSIS', 'SYNCOPE', 'TACHYCARDIA', 'PALPITATIONS', 'THROAT TIGHTNESS', 'BLOOD PRESSURE INCREASED', 'ANAPHYLACTIC REACTION', 'LOSS OF CONSCIOUSNESS', 'DECREASED APPETITE', 'MUSCULAR WEAKNESS', 'MOBILITY DECREASED', 'FEELING HOT', 'INJECTION SITE SWELLING', 'SARS- COV-2 TEST POSITIVE']

**Table 5:** The most significant features within the patient adverse reactions after MODERNA vaccination for each target class.

No. of selected features		Selected features
Died	36	['AGE_YRS', 'L_THREAT', 'PRIOR_VAX', 'OFC_VISIT', 'ER_ED_VISIT', 'ALLERGIES', 'VAX_DOSE_SERIES', 'HEADACHE', 'DYSPNOEA', 'FATIGUE', 'CHILLS', 'NAUSEA', 'COVID-19', 'MALAISE', 'COUGH', 'MYALGIA', 'CONDITION AGGRAVATED', 'PARAESTHESIA', 'PRURITUS', 'FACIAL PARALYSIS', 'PALPITATIONS', 'HYPERHIDROSIS', 'ERYTHEMA', 'THROAT TIGHTNESS', 'TREMOR', 'BLOOD PRESSURE INCREASED', 'INTENSIVE CARE', 'LOSS OF CONSCIOUSNESS', 'MUSCULAR WEAKNESS', 'FLUSHING', 'MOBILITY DECREASED', 'FEELING HOT', 'INJECTION SITE SWELLING', 'CEREBROVASCULAR ACCIDENT', 'CARDIAC ARREST', 'SARS-COV-2 TEST POSITIVE']
Hospitalized	34	['AGE_YRS', 'SEX', 'L_THREAT', 'OTHER_MEDS', 'BIRTH_DEFECT', 'OFC_VISIT', 'ER_ED_VISIT', 'ALLERGIES', 'VAX_DOSE_SERIES', 'DISABLE', 'HEADACHE', 'PYREXIA', 'NAUSEA', 'COVID-19', 'PAIN IN EXTREMITY', 'COUGH', 'HYPOAESTHESIA', 'CHEST PAIN', 'RASH', 'PARAESTHESIA', 'PRURITUS', 'URTICARIA', 'FACIAL PARALYSIS', 'SYNCOPE', 'TACHYCARDIA', 'HYPERHIDROSIS', 'ERYTHEMA', 'TREMOR', 'INTENSIVE CARE', 'DECREASED APPETITE', 'INJECTION SITE ERYTHEMA', 'CARDIAC ARREST', 'LYMPHADENOPATHY', 'SARS-COV-2 TEST POSITIVE']
Recovered	29	['SEX', 'L_THREAT', 'BIRTH_DEFECT', 'OFC_VISIT', 'ER_ED_VISIT', 'ALLERGIES', 'VAX_DOSE_SERIES', 'HEADACHE', 'CHILLS', 'PAIN', 'DIZZINESS', 'PAIN IN EXTREMITY', 'ASTHENIA', 'HYPOAESTHESIA', 'FEELING ABNORMAL', 'CHEST DISCOMFORT', 'ARTHRALGIA', 'PARAESTHESIA', 'UNRESPONSIVE TO STIMULI', 'PRURITUS', 'HEART RATE INCREASED', 'URTICARIA', 'TACHYCARDIA', 'THROAT TIGHTNESS', 'ANAPHYLACTIC REACTION', 'LOSS OF CONSCIOUSNESS', 'FLUSHING', 'INJECTION SITE SWELLING', 'CARDIAC ARREST']

### Prediction phase

**Figure 5:**
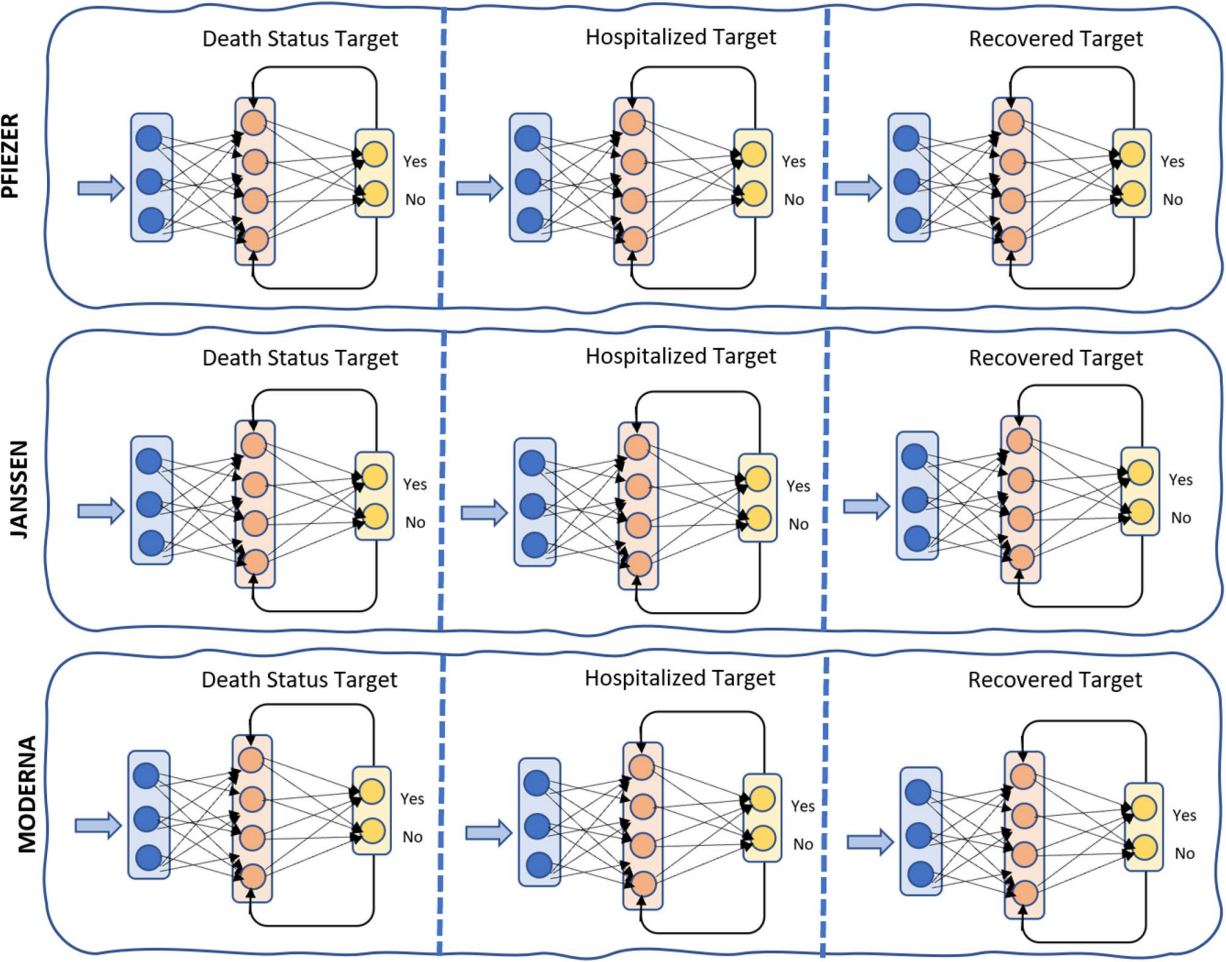
RNNs for the three target classes for every vaccine type.

In the proposed model, three target classes “Death Status”, “Hospitalized”, and classes “Recovered” are predicted, it should be noted that the classes are not mutually exclusive, meaning that more than one target class can occur at the same recipient. For example, a vaccine recipient could get hospitalized and then die, or get hospitalized and then recoverd. So, one target class is predicted at a time with each vaccine type, as described in Fig. 5. For each vaccine (PFIEZER, JANSSEN, and MODERNA), the three target classes “Death Status”, “Hospitalized”, and classes “Recovered” are predicted one at time. That is, the “Death Status” target class for PFIEZER vaccine is predicted, “Hospitalized” target class for PFIEZER vaccine is predicted, and finally the “Recovered” target class for PFIEZER vaccine is predicted. The same ais done for JANSSEN, and MODERNA vaccines. In other words, if PFIEZER is taken by a recipient, how likely this recipient will die, hospitalized, or recovered.

## Experimental results

This section provides the findings of this research. The experiments were conducted on a 3 GHz i5 computer with a 4 GB main memory and 64-bit Windows 7 operating system. The experiment is carried out using the python programming language.

### Features intersection among the three vaccines for the patient’s target class

From a pharmacovigilance perspective for studied COVID-19 vaccines, the purpose of this study was to discover the most common side effects are common in each patient category.

**Figure 6:**
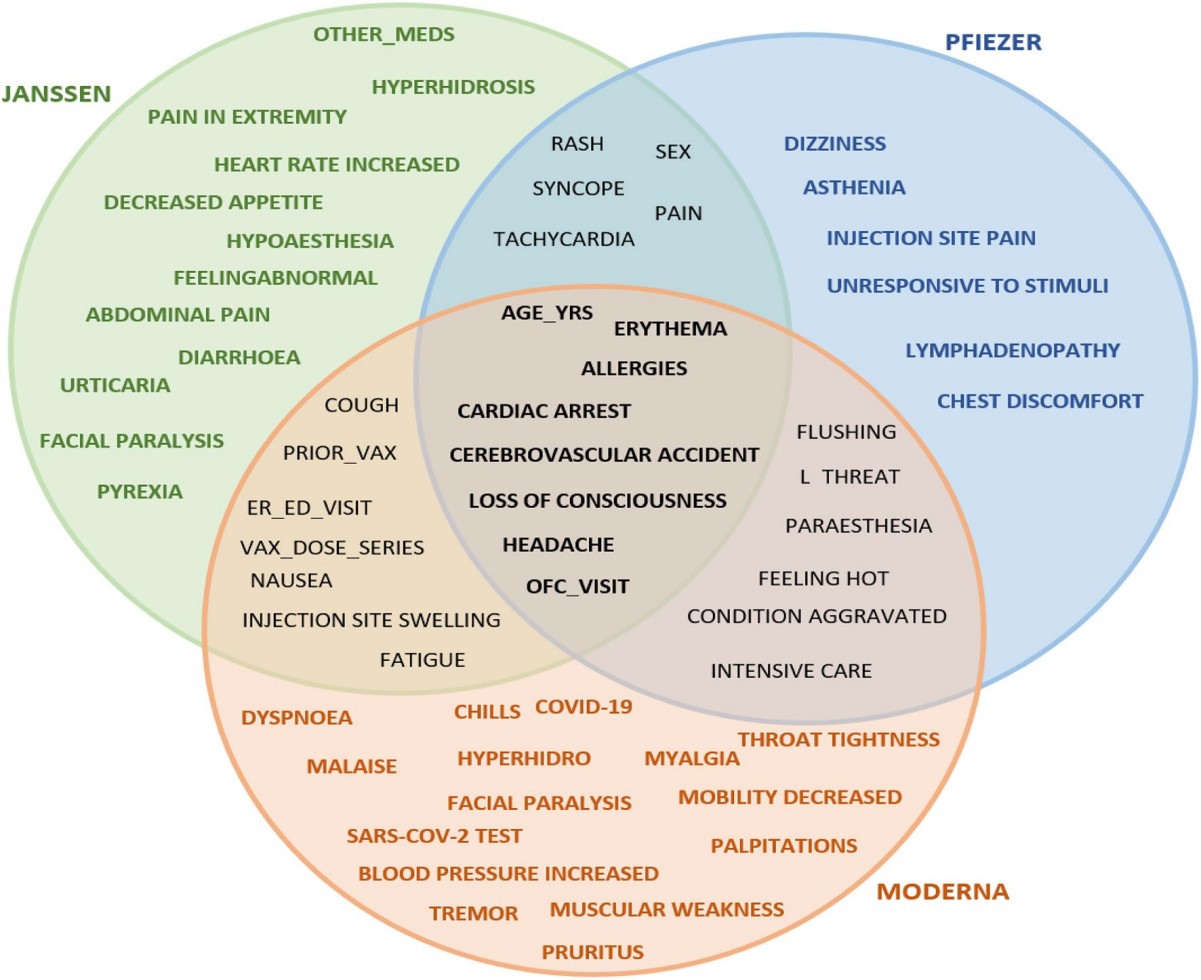
Features intersection among the three vaccines for the “Death” patients target class.

**Figure 7:**
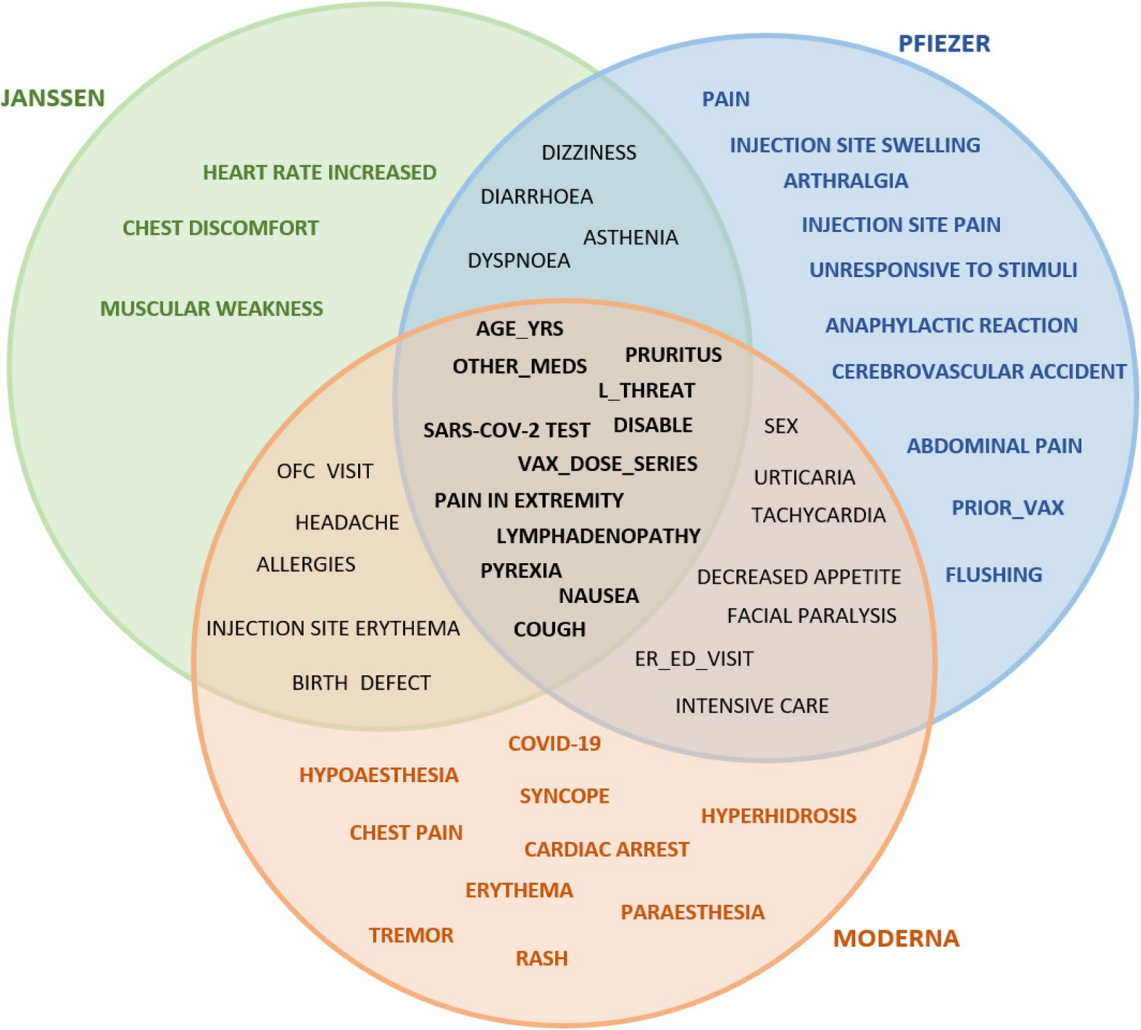
Features intersection among the three vaccines for the “Hospitalized” patients’ class.

**Figure 8:**
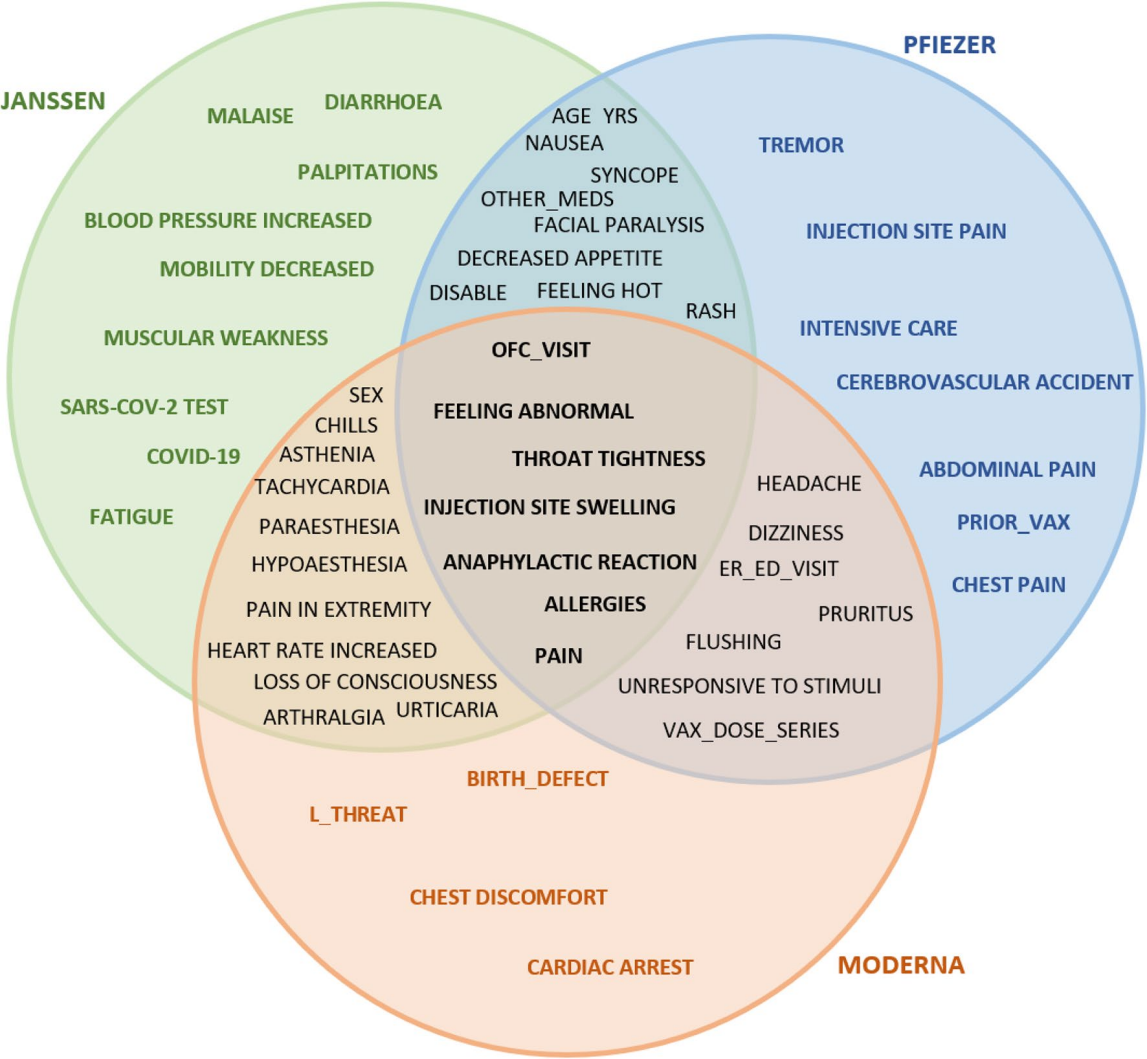
Features intersection among the three vaccines for the “Recovered” patients’ target class.

Figures 6, 7, 8 showed feature intersections among the three vaccines for patient’s classes “Death”, “Hospitalized”, and “Recovered” respectively. It can be noticed from the figures that some features are common between the three vaccines while others are specified for each one of them.

In the “Death” class, it is found that the three vaccines share seven features (age_yrs, erythema, allergies, cardiac arrest, cerebrovascular accident, loss of consciousness, and ofc_visit), while the chest discomfort feature, for example, is specific for only the PFIZER vaccine.

### Relation between side effects for each category after injection of the COVID-19 vaccine

**Figure 9:**
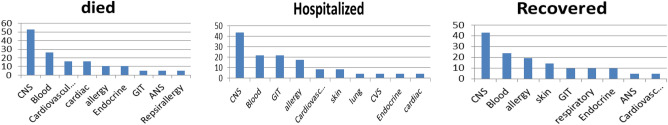
No of side effects based on their effect in organs in died, hospitalized, and recovered patients after PFIEZER vaccine.

**Figure 10:**
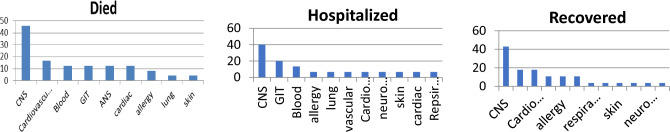
No of side effects based on their effect in organs in died, hospitalized, and recovered patients after JANSSEN vaccine.

**Figure 11:**
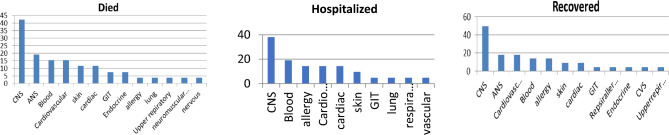
No side effects based on their effect on organs in died, hospitalized, and recovered patients after MODERNA vaccine.

Figures 9, 10 and 11 show the number of side effects based on their effect on organs in died, hospitalized, and recovered patients after the three vaccines for each class. In the case of PFIEZER vaccine, the CNS-related side effects are the most common in all patient categories than blood-related side effects. The third most common side effects in died, hospitalized, and recovered people are CVS, GIT, and allergy side effects respectively, Fig. 9. in the case of JANSSEN vaccine, the CNS-related side effects are the most common in all patient categories then CVS-related side effects in case of died and recovered categories, Fig. 10.

In the case of MODERNA vaccine, the CNS-related side effects are the most common in all patient categories than ANS-related side effects in death and recovery. In cases hospitalized, blood is the second most common side effect. The third common side effect in dead, and recovered people is cardiovascular side-related side effects, Fig. 11.

### Classification of patients using deep learning classifier

Among various types of deep learning models, recurrent neural networks (RNNs) and long short-term memory (LSTM) networks have gained significant attention for their ability to handle data. In this section, we will compare between RNNs and LSTMs models for the classification of patients using a deep learning classifier. We will explore the strengths and limitations of each model and highlight the factors and different performance evaluation metrics like (Accuracy, Recall, Specificity, Precision, F1_Score and computational time. Tables 6, 7 and 8 are show comparison between RNNs and LSTMs models for the classification of patients’ adverse reactions after the three vaccines of the three target classes “Death Status”, “Hospitalized”, and “Recovered”.

**Table 6:** Comparison between RNNs and LSTMs models for the patients’ adverse reactions after MODERNA vaccine and in three target classes using different performance evaluation metrics.

MODERNA									
Model	Hospitalized			Recoved			Died		
	Accuracy	Recall	Specificity	Accuracy	Recall	Specificity	Accuracy	Recall	Specificity
RNN	92.704	97.027	88.536	97.794	97.473	97.981	93.485	96.582	90.456
	Precision	F1_Score	Time	Precision	F1_Score	Time	Precision	F1_Score	Time
	89.084	92.886	3025.82	96.574	97.021	604.277	90.826	93.615	5154.79
LSTM	92.09	89.49	94.12	82.82	72.01	95.83	89.17	95.09	84.79
	Precision	F1_Score	Time	Precision	F1_Score	Time	Precision	F1_Score	Time
	92.29	90.87	484.630	95.43	82.08	166.185	82.00	88.06	687.35

**Table 7:** Comparison between RNNs and LSTMs models for the patients’ adverse reactions after PFIZER vaccine and in three target classes using different performance evaluation metrics.

PFIZER									
Model	Hospitalized			Recoved			Died		
	Accuracy	Recall	Specificity	Accuracy	Recall	Specificity	Accuracy	Recall	Specificity
RNN	94.161	94.622	93.716	81.627	84.263	79.212	96.031	96.931	95.315
	Precision	F1_Score	Time	Precision	F1_Score	Time	Precision	F1_Score	Time
	93.572	94.094	4645.51	78.789	81.434	1628.154	94.277	95.586	4345.362
LSTM	89.14	75.80	97.90	83.07	81.6	84.09	90.33	65.30	97.99
	Precision	F1_Score	Time	Precision	F1_Score	Time	Precision	F1_Score	Time
	95.95	84.70	703.027	78.15	79.84	303.792	90.91	76.01	720.69

**Table 8:** Comparison between RNNs and LSTMs models for the patients’ adverse reactions after JANSSEN vaccine and in three target classes using different performance evaluation metrics.

JANSSEN									
Model	Hospitalized			Recoved			Died		
	Accuracy	Recall	Specificity	Accuracy	Recall	Specificity	Accuracy	Recall	Specificity
RNN	94.7	92.2	97.10	85.96	92.20	77.251	93.56	93.01	94.01
	Precision	F1_Score	Time	Precision	F1_Score	Time	Precision	F1_Score	Time
	96.84	94.46	330.406	85	88.45	269.44	92.69	92.85	512.089
LSTM	94.48	90.72	97.02	74.46	77.27	71.48	81.38	83.72	79.77
	Precision	F1_Score	Time	Precision	F1_Score	Time	Precision	F1_Score	Time
	95.39	92.99	46.102	74.18	75.69	50.154	73.99	78.55	91.365

Above tables and the results show that RNN model outperformed LSTM in several metrics such as accuracy, recall, specificity, precision, and F1 score, indicating its superior performance in patient classification tasks. On the other hand, the RNN model is worse than the LSTM with respect to the computational time and this limitation will be considered as a research point for future research.

### Classification of patients using RNN classifier

**Figure 12:**
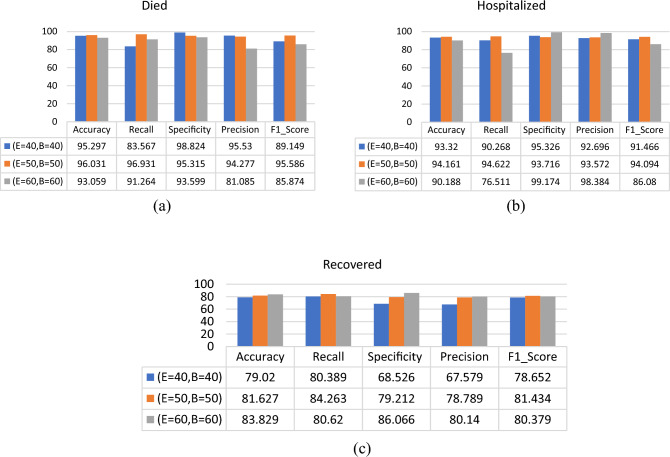
Performance comparison for the patients’ adverse reactions after PFIEZER vaccine using different number of epochs and different patch size where E represent number of epochs and B represent batch size. (**a**) classification of died patients. (**b**) classification of hospitalized patients. (**c**) classification of recovered patients.

**Figure 13:**
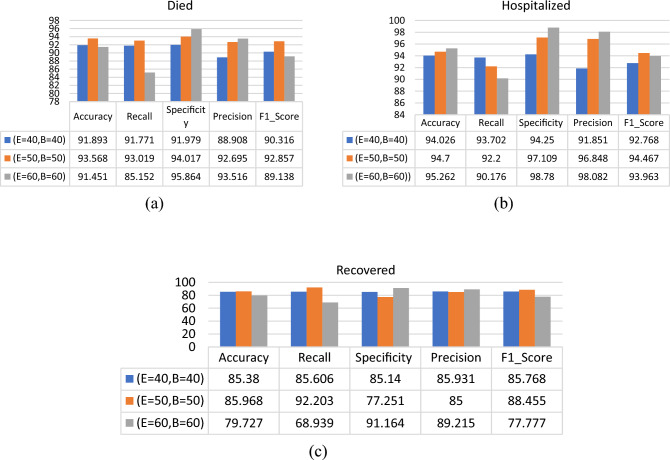
Performance comparison for the patients’ adverse reactions after JANSSEN vaccine using a different number of epochs and different patch size where E represents the number of epochs and B represent the batch size. (**a**) classification of dead patients. (**b**) classification of hospitalized patients. (**c**) classification of recovered patients.

**Figure 14:**
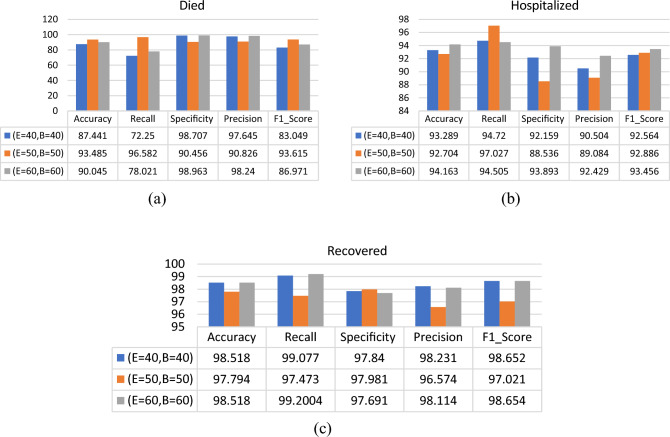
Performance comparison for the patients’ adverse reactions after MODERNA using a different number of epochs and different patch size where E represents the number of epochs and B represent the batch size. (**a**) classification of dead patients. (**b**) classification of hospitalized patients. (**c**) classification of recovered patients.

The RNN classifier has been tested using different values for batch size and epochs. Figures 12, 13 and 14 show the performance comparison for the patients’ adverse reactions after the three vaccines in terms of accuracy, recall, specificity, precision, and the F1_score of the three target classes “Death Status”, “Hospitalized”, and “Recovered”. it is observed that the proposed model performance was better in most cases when using 50 epochs and the batch size was equal to 50. Accordingly, the best parameters used for the RNN classifier are given in Table 6.

**Figure 15:**
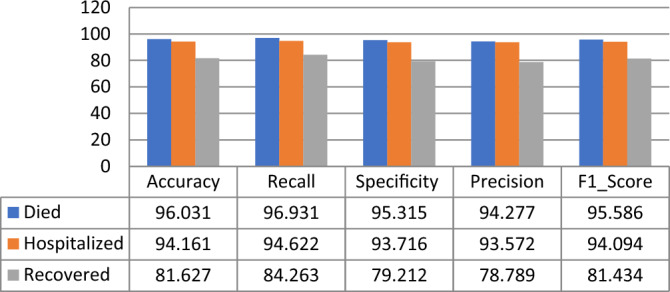
Performance comparison for the patients’ adverse reactions after PFIEZER vaccination for each class.

**Figure 16:**
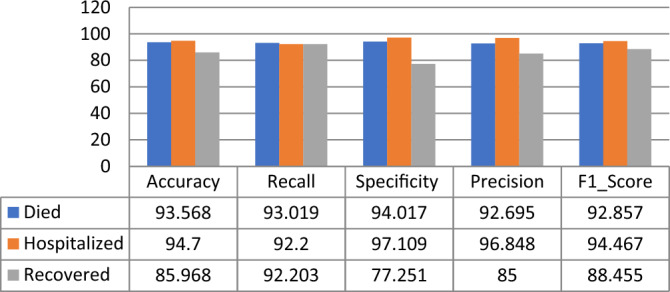
Performance comparison for the patients’ adverse reactions after JANSSEN vaccination for each class.

**Figure17:**
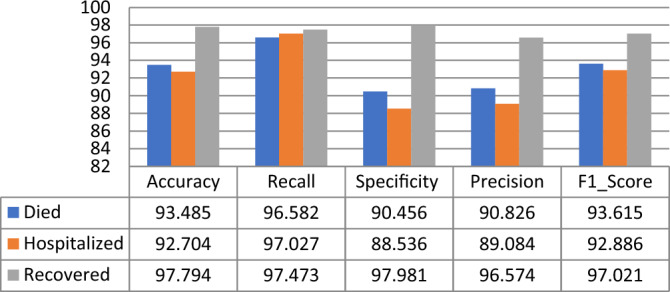
Performance comparison for the patients’ adverse reactions after MODERNA vaccination for each class.

Using the RNN classifier parameters given in Table 9, it is observed that the proposed model gives the highest Accuracy, Recall, F1_Score, Specificity, and Precision scores for the “Death Statues” class target in PFIEZER vaccination with an accuracy value of 96.03% as shown in Fig. 15. While in JANSSEN vaccination, Fig. 16 showed that the “Hospitalized” target class has highest performance with accuracy reading 94.7%. And finally, the model has the best performance for the “Recovered” class in the MODERNA vaccination with an accuracy of 97.794% as shown in Fig. 17.

**Table 9:** The best parameters used for the recurrent neural network classifier.

Parameter	Value
Batch size	50
Number of epochs	50
Activation function	Sigmoid’

**Figure 18:**
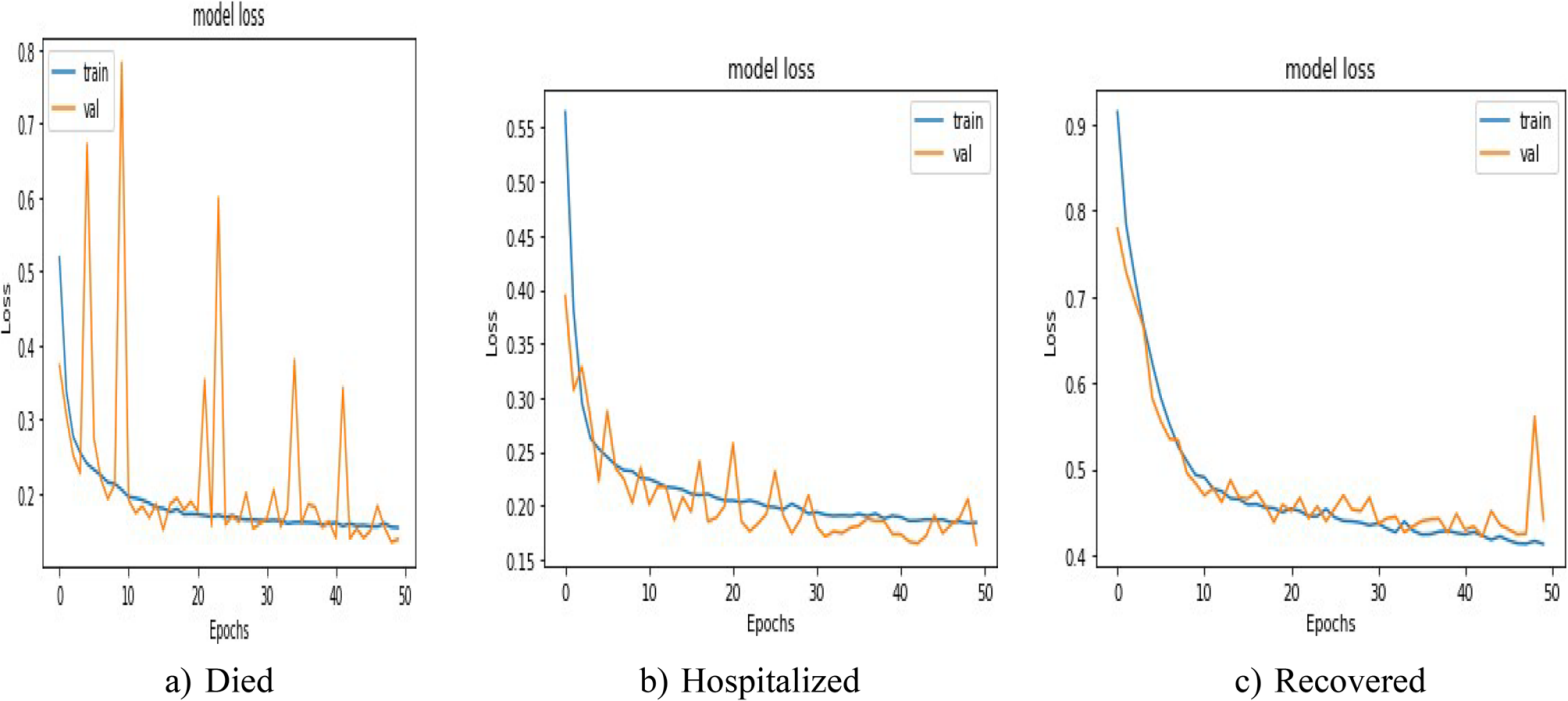
Loss comparison for the validation and training datasets of the patients’ adverse reactions after PFIEZER vaccine. (**a**) model loss for died patients. (**b**) model loss for hospitalized patients. (**c**) model loss for recovered patients.

**Figure 19:**
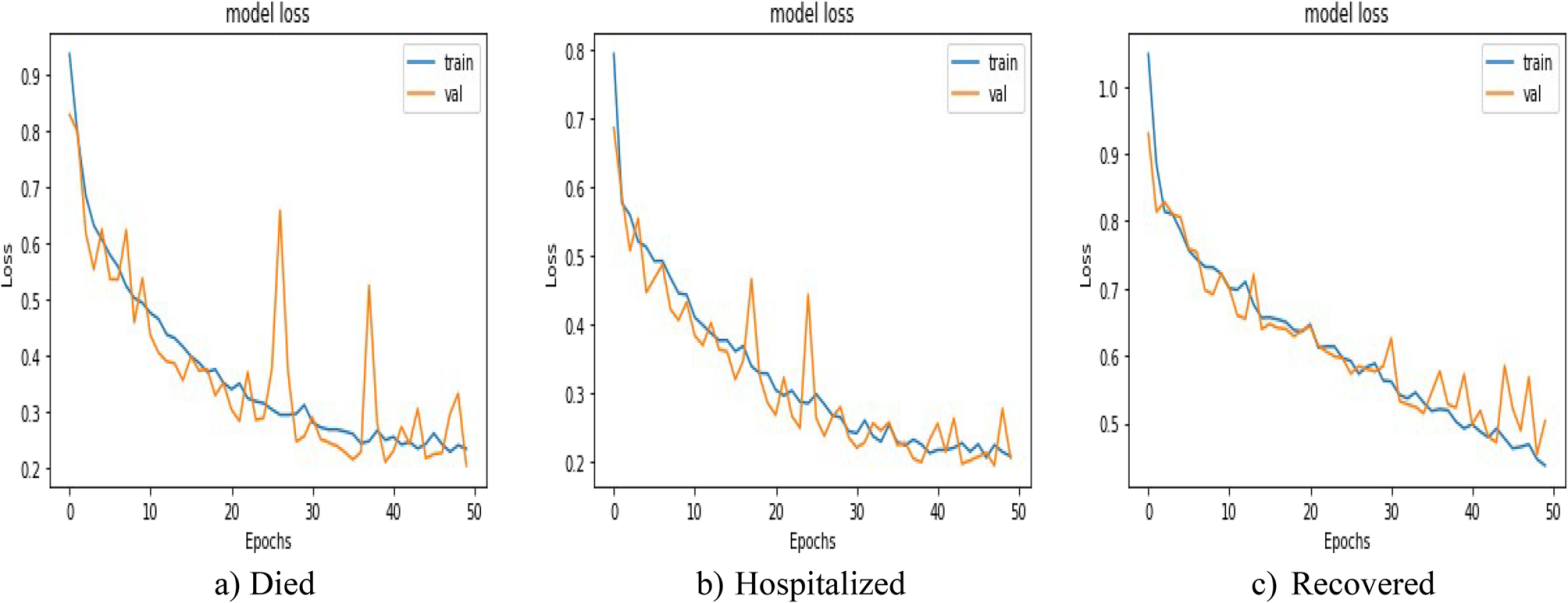
Loss comparison for the validation and training datasets of the patients’ adverse reactions after JANSSEN vaccine. (**a**) model loss for died patients. (**b**) model loss for hospitalized patients. (**c**) model loss for recovered patients.

**Figure 20:**
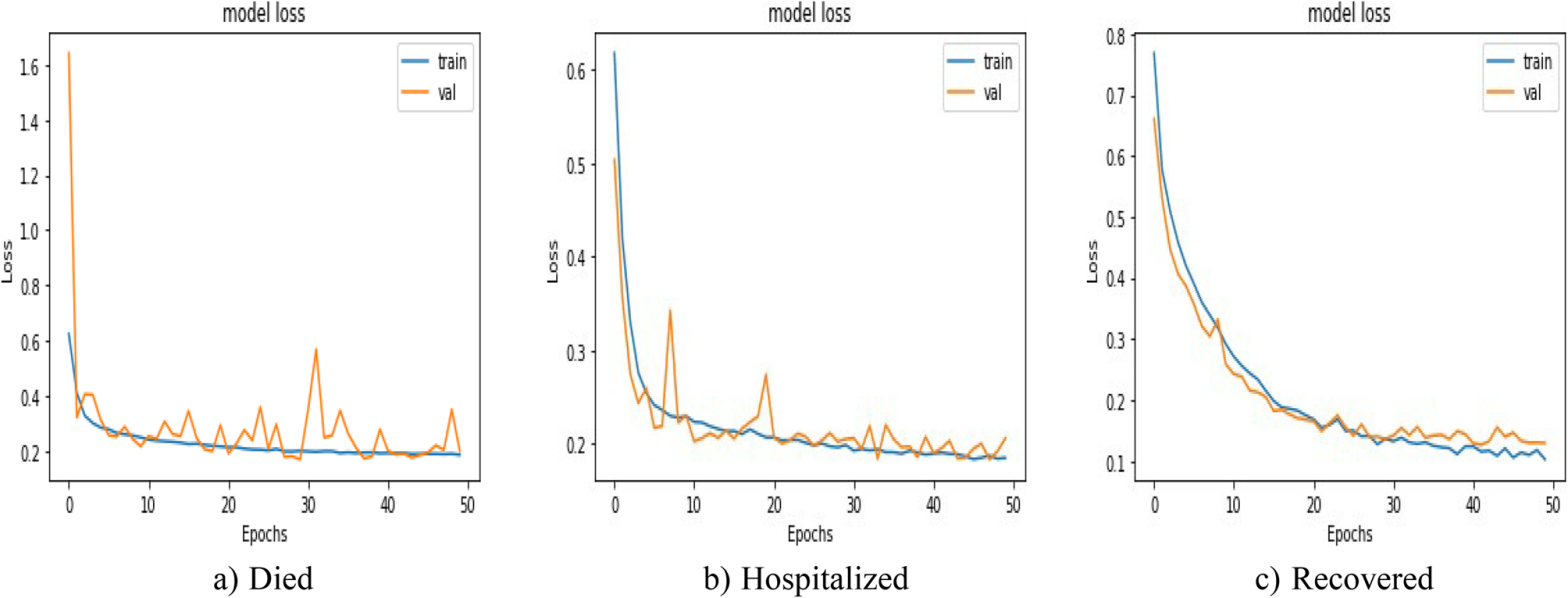
Loss comparison for the validation and training datasets of the patients’ adverse reactions after MODERNA vaccine. (**a**) model loss for died patients. (**b**) model loss for hospitalized patients. (**c**) model loss for recovered patients.

**Figure 21:**
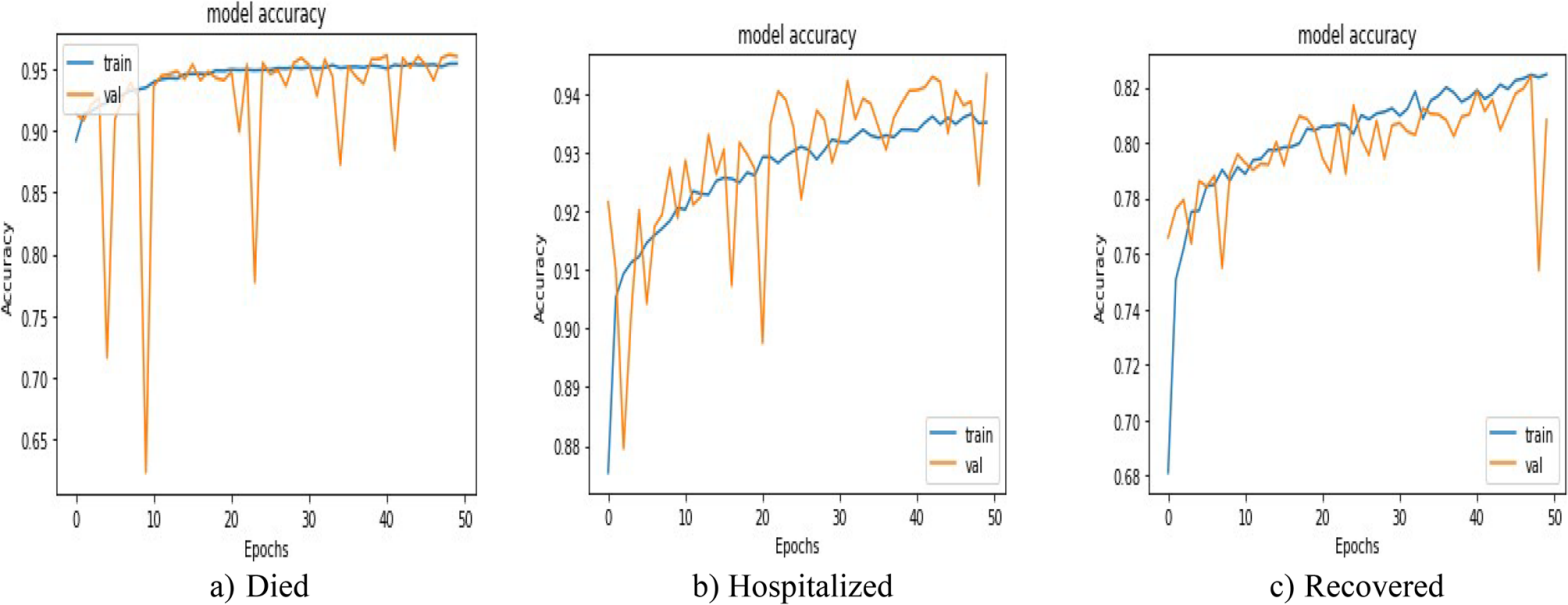
Accuracy comparison for the validation and training datasets of the patients’ adverse reactions after PFIEZER vaccine. (**a**) accuracy for died patients. (**b**) model accuracy for hospitalized patients. (**c**) model accuracy for recovered patients.

**Figure 22:**
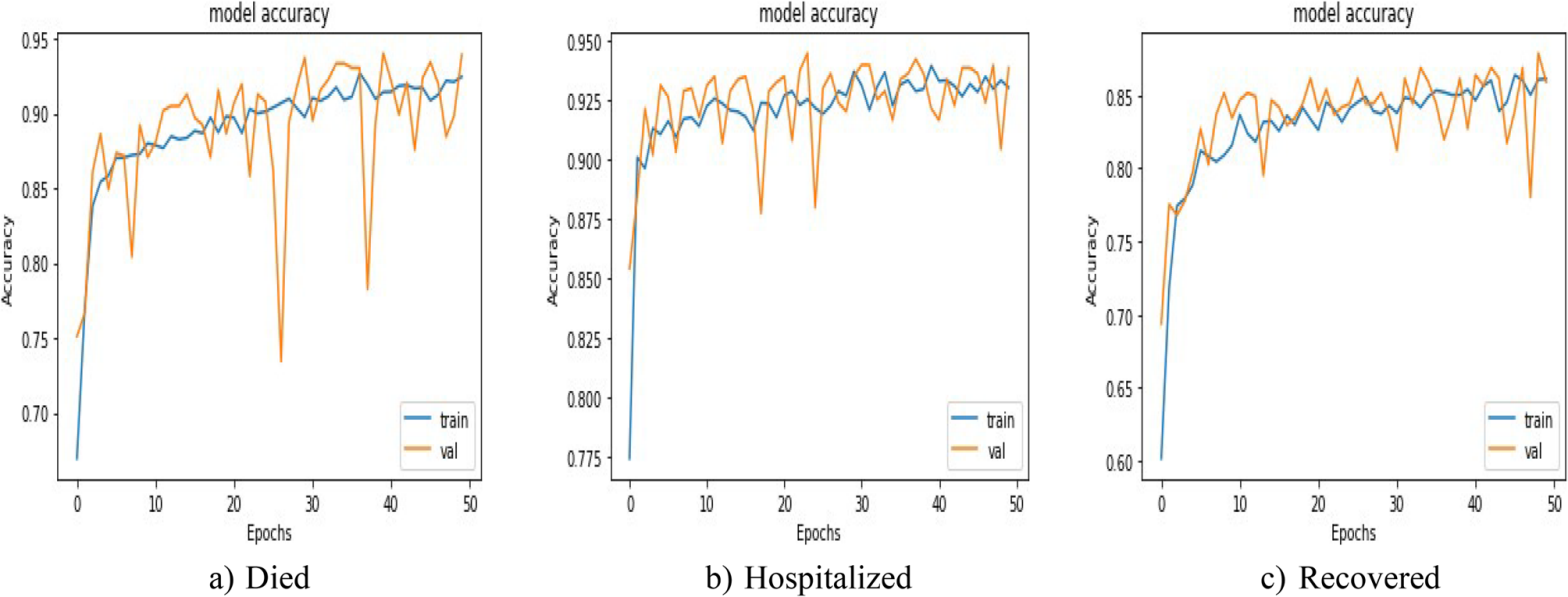
Accuracy comparison for the validation and training datasets of the patients’ adverse reactions after JANSSEN vaccine. (**a**) accuracy for dead patients. (**b**) model accuracy for hospitalized patients. (**c**) model accuracy for recovered patients.

**Figure 23:**
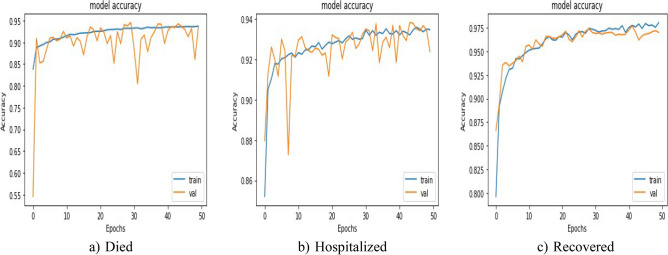
Accuracy comparison for the validation and training datasets of the patients’ adverse reactions after MODERNA vaccine. (**a**) accuracy for dead patients. (**b**) model accuracy for hospitalized patients. (**c**) model accuracy for recovered patients.

Loss comparison for the validation and training datasets of the patients’ adverse reactions after PFIEZER, JANSSEN, and MODERNA vaccination for each class is given in Figs. 18, 19, and 20 respectively. A similar Accuracy comparison for the validation and training datasets of the patients’ adverse reactions after PFIEZER, JANSSEN, and MODERNA vaccination for each class is given in Figs. 21, 22, and 23 respectively.

Last but not least, with the development of vaccines with a limited initial supply, such methods may be useful in identifying high-risk patients for primary vaccination campaigns. Educating the general public about vaccine safety is critical to public health and ongoing and future large-scale vaccination campaigns. The obtained results will help in pharmacovigilance and drug safety approaches to choose the best vaccine based on the medical history of the patient.

### Statistical analysis

A Wilcoxon Signed Rank test was conducted to determine if there is a statistically significant difference between the proposed model using RNN and the LSTM model. The Wilcoxon Signed Rank test is a non-parametric statistical test used to compare two related samples. It is commonly used to determine whether there is a significant difference between two methods or treatments applied to the same group of subjects. The test is particularly useful when the assumption of normality is not met, or when the sample size is small. The null hypothesis of the test is that there is no difference between the population medians of the two samples, and the alternative hypothesis is that the medians are not equal. If the p-value is less than the significance level (usually 0.05), the null hypothesis is rejected, and it is concluded that there is a significant difference between the two samples^31^. The descriptive statistics for the two models showed that RNN had a higher mean accuracy than the LSTM model where the mean accuracy of the LSTM model was 86.197778 (SD = 6.2431678) and the mean accuracy of the RNN meodel was 91.941778 (SD = 4.9613537) as showm in Table 10.

**Table 10:** Descriptive statistics for the RNN and LSTM models.

	Mean	Std. deviation	Minimum	Maximum
LSTM	86.197778	6.2431678	74.4600	94.4800
RNN	91.941778	4.9613537	81.6270	97.7940

The Wilcoxon signed-rank test indicated that the RNN model had a higher mean rank (mean rank = 3.00) than the LSTM model, indicating a significant difference between the two models. Moreover, there was a significant difference in accuracy between the LSTM and RNN models (Z = − 2.312, p = 0.021 two-tailed). The negative Z-value suggests that Accuracy_RNN is statistically significantly lower than Accuracy_Lstm. The p-value of 0.021 indicates that there is a 2.1% chance of observing such a large difference between the two models by chance alone, and that this difference is statistically significant at the 0.05 level. Table 11: Summarize the results of Wilcoxon signed-rank test for the RNN and LSTM models.

**Table 11:** Summary of Wilcoxon signed-rank test results the RNN and LSTM models.

Model	Ranks		Test statistics	
	Mean ranks	Sum of ranks	Z-score	p-value
RNN	3.00	3.00	− 2.312	0.021
LSTM	5.25	42.00		

## Conclusion

In this paper, a DL-based model has been developed to study the adverse reactions of Covid-19 post-vaccination of three vaccines (PFIEZER, JANSSEN, and MODERNA). Only three categories are considered, Death status, Hospitalized, and Recovered.

Based on the accuracy obtained, we can conclude that the proposed model is a promising model for identifying the relationship between the type of COVID-19 vaccine and the side effects that appear on patients after vaccination.

Based on the work done, some key points are summarized as follows:

- It can be inferred that certain side effects were increased in patients according to the type of COVID-19 vaccines.
- Side effects related to CNS and hemopoietic model were high in all types of COVID-19 vaccine.
- The analysis of the ratio of side effects that were detected in hospitalized people more than the recovered people according to the type of COVID-19 vaccine.
- The number of allergies and cardiovascular side effects increase after vaccination with PFIZER vaccine.
- The number of GIT, and blood side effects increase after vaccination with JANSSEN vaccine.
- The number of blood and allergy side effects increase after vaccination with MODERNA vaccine.

As illustrated previously, the literature is very rich in COVID-19 research and its correlation to different scientific fields. However, most of the work, especially those involving AI applications, was concerned with the prediction or the diagnosis of COVID-19, either by using the current condition and symptoms of the patient as features or the chest X-ray to diagnose the disease. To the best of our knowledge, this is the first research that uses DL to predict the adverse effects a patient may experience after the COVID-19 vaccination, which will have a remarkable impact on the public health concerns related to COVID-19 vaccine, either on the level of taking a primary dose, taken a booster dose of the already approved vaccines or for those still under development in the pre-clinical, first, second, and third vaccine development phases and will be introduced in the nearest future.

The necessity to get more information about COVID-19 is still required. The future suggested work to identify the relationship between side effects generated from COVID-19 vaccines and other approved WHO vaccines to minimize the severity of the administration of both therapies in a specific patient. Applying another DL model to the investigated problem with a satisfiable computational time may enhance the prediction accuracy and can be considered as a new point for future research.

## Data Availability

The datasets generated and/or analyzed during the current study are available in the [**vaers**] repository, [https://vaers.hhs.gov/data/datasets.html]. There is No human or animal subject used in this study. The data is public data.
